# Education of Children

**Published:** 1871-04

**Authors:** 


### EDUCATION OF CHILDREN.

We have been reminded upon several occasions recently, of the disastrous effects that our present system of education has wrought upon the health and development of our children, and have ventured to suggest a reform, whereby much suffering could be averted, and the object of teaching better secured. Our school laws should be so amended as to exclude children from the school room until they have attained a certain age. At present, the teachers of our public schools are compelled to receive, and must attempt to instruct, children who should be under the watchful care of the mother. Many parents send their little ones to school in order to have the teacher act the part of a nurse, in overseeing and keeping them out of danger. Children at four and five years of age, are utterly incapable of fixing their minds upon a lesson, for any length of time. Such a task should not be required of them,—it is of far more importance that their physical development should be encouraged, for the mental faculties will grow in proportion to the amount of strength furnished from a full and vigorous body. A child, at this age, will not bear confinement, it is not calculated to sit six hours upon a hard seat, without any liberty of motion, its eye directed upon a book, trying to solve those mysteries which its brain is not yet prepared to receive. The child requires exercise in the open air—liberty to run, tumble and scream, in order that its vital organs may become developed, when, by play and observation, it soon becomes both bodily and mentally, capable of directing its attention to the contemplation of exercises calculated to develop the mind and intellect.

The child should receive its elementary education at home—should be required to recite twice a day, but should not be detained more than one hour in the morning, and about the same length of time in the afternoon. The balance of the day should be allowed him for free and boisterous play, in the open air. Not the least danger is imminent of the child appearing awkward and unintelligent, if kept from school until he has reached the age of eight or nine years, if the parents are only careful to admit him to the parlor during the visitations of older persons; children are great imitators, and acquire a fund of information by observation. They will learn more while at play, up to eight years of age, than you could possibly *cram* into them while imprisoned in the school room. This fact is' observable in every school, where it is found, with very rare exceptions, the child who has been *driven* to school when quite young, and there kept in confinement from day to day, when every instinct and desire of its nature is for liberty, that it is a very dull scholar, when compared with those of the same age who commenced study at a later period, and who had the advantage of play and exercise when the other was confined.

As the child grows older and exhibits evidence of improvement in its mental energies, so should the studies be gradually multiplied, being always careful not to overtax the talent of the student. The system of graded schools, as adopted in our own city, is an excellent training for the young mind, if parents are only careful to allow the teacher to judge when the scholar is qualified to enter a higher grade. Most parents are impatient to have their children rise rapidly—to make an exhibition of their marvelous progress, and so urge the teacher to advance them before they are qualified for such a position. This is a fatal mistake, and often suicidal to the health and intelligence of the child. Our children are urged too much,—become mature far in advance of their time,—complete their education and are rushed into business and manhood while they are yet, or should be, children. For this reason, more than any other, are we a sickly, puny, short-lived race. We must give our children more play, begin their education at a later period, so that it shall not be completed until they shall have attained the age of twenty-five, when they will be better qualified, mentally and physically, to take upon themselves the sterner duties of life.

